# Different genotypes and species of symbiotic fungi mediate the behavioral response of invasive *Sirex noctilio* fabricius (Hymenoptera: Siricidae)

**DOI:** 10.3389/fmicb.2024.1341646

**Published:** 2024-07-11

**Authors:** Ming Wang, ChengLong Gao, QinWang Xu, NingNing Fu, JiaLe Li, LiLi Ren, YouQing Luo

**Affiliations:** ^1^Beijing Key Laboratory for Forest Pest Control, Beijing Forestry University, Beijing, China; ^2^Guangdong Key Laboratory of Animal Conservation and Resource Utilization, Guangdong Public Laboratory of Wild Animal Conservation and Utilization, Institute of Zoology, Guangdong Academy of Sciences, Guangzhou, China; ^3^Sino-French Joint Laboratory for Invasive Forest Pests in Eurasia, INRAE-Beijing Forestry University, Beijing, China; ^4^Guangdong Provincial Key Laboratory of Silviculture, Protection and Utilization, Guangdong Academy of Forestry, Guangzhou, China; ^5^Department of Forest Protection, College of Forestry, Hebei Agricultural University, Baoding, China

**Keywords:** *Sirex* woodwasp, volatiles, olfactory assays, oviposition, symbiotic fungi

## Abstract

In northeast China, the invasive woodwasp., *Sirex noctilio*, attacks *Pinus sylvestris* var. *mongolica* Litv and often shares habitat with native *Sirex nitobei*. Previous research showed that *S. noctilio* can utilize the volatiles from its symbiotic fungus (*A. areolatum* IGS-BD) to locate host trees. Consequently, symbiotic fungi (*A. areolatum* IGS-D and *A. chailletii*) carried by *S. nitobei* may influence the behavioral selection of *S. noctilio*. This study aimed to investigate the impact of fungal odor sources on *S. noctilio*’s behavior in laboratory and field experiments. Our observations revealed that female woodwasps exhibited greater attraction toward the fungal volatiles of 14-day-old *Amylostereum* IGS-D in a “Y”-tube olfactometer and wind tunnel. When woodwasps were released into bolts inoculated separately with three strains in the field, females of *S. noctilio* exhibited a preference for those bolts pre-inoculated with *A. areolatum* IGS-BD. Gas chromatography–mass spectrometry (GC–MS) analysis revealed that the volatiles emitted by the two genotypes of *A. areolatum* were similar yet significantly distinct from those of *Ampelopsis chailletii*. Hence, we postulate that the existence of native *A. areolatum* IGS-D could potentially facilitate the colonization of *S. noctilio* in scenarios with minimal or no *A. areolatum* IGS-BD present in the host.

## 1 Introduction

*Sirex noctilio* Fabricius (Hymenoptera: Siricidae), native to Europe and northern Africa, is a major pest in pine forests (Spradbery, 1973). It has accidentally invaded Oceania, Africa, and South America in the past century, and more recently, North America and northeast China through the plantations of exotic pines and human activities (Talbot, 1977; Spradbery and Kirk, 1978; Madden, 1988; Li et al., 2015; Sun et al., 2016). Host species *S. noctilio* include *Pinus*, *Abies*, *Larix* and *Picea* species etc. (Madden, 1988). *Sirex noctilio* has colonized only *P. sylvestris* var. *mongolica* Litv in northeast China (Li et al., 2015; Sun et al., 2016). Wasps deposit a combination of eggs, venom gland secretions, and the mutualist fungus *Amylostereum* via drills into the sapwood (Kobayashi et al., 1978; Fukuda and Hijii, 1996; Cooperband et al., 2012; Ryan et al., 2012b; Wang et al., 2017; Gao et al., 2022). The fungal mutualists can grow in host trees when the wasps are absent, although they usually depend on wasps for long-range dispersal (Wermelinger and Thomsen, 2012; Wang et al., 2020).

The process of oviposition by insect herbivores typically involves host location, followed by acceptance. Host location is primarily mediated by olfaction and vision, starting from a distance. Adult woodwasps exhibit a brief lifespan, necessitating the execution of mating and spawning behaviors within a distinctly limited timeframe (approximately 7 ± 4 days for males and 7 ± 3 days for females) (Fukuda et al., 1993; Slippers et al., 2012). Any delay in host location by 1–2 days significantly impacts their reproduction (Hajek et al., 2021). Consequently, it is hypothesized that *S. noctilio* exhibits a potent selection ability for promptly identifying hosts. Host plant volatiles, particularly monoterpenes, play a crucial role in attracting woodwasps to host trees over long distances (Madden, 1988; Slippers et al., 2015; Xu et al., 2019b). Monomers or blends of monoterpenes are commonly utilized in field pest monitoring to attract females (Erbilgin et al., 2002; Bashford, 2008; Batista et al., 2018). Additionally, fungal volatiles provide reliable cues for *S. noctilio* in assessing suitable hosts (Titze, 1965; Ryan et al., 2012a; Sarvary et al., 2016). Volatiles emitted by fungal symbionts seem to exert a stronger attractant effect compared to those produced by the host (Fernandez Ajo et al., 2015; Sarvary et al., 2016). A strong synergistic effect was observed between the volatiles emitted by the symbiotic fungi and those emitted by the host trees (Faal et al., 2021; Masagué et al., 2023).

Research has shown that at low population densities, *S. noctilio* preferentially selects relatively weak host trees, as these hosts are more conducive to the growth and development of their offspring (Slippers et al., 2012). Meanwhile, other bark beetles and wood borers are likely to infest weakened hosts, which, along with their fungal mutualists, can negatively impact both the availability and suitability of host trees for *S. noctilio* (Ryan et al., 2012a; Foelker, 2016; Wang et al., 2018). To avoid nutrient competition between populations, *S. noctilio* colonizes by avoiding locations where other insects lay their eggs (Wang, 2017; Xu et al., 2019b). Ryan discovered that *S. noctilio* avoided drilling into wood inoculated with the ophiostomatoid fungus *Leptographium wingfieldii*, a fungus vectored by bark beetles (Ryan and Hurley, 2012; Ryan et al., 2012a), confirming that symbiotic fungi associated with other insects can impact the selection of oviposition sites by *S. noctilio*. On the other hand, the endophytic fungi of host plants can also impact the selection of oviposition sites by *S. noctilio*. Infection of the host tree by *Ophiostoma* inhibits the growth of *A. areolatum*, thereby indirectly impacting the development of woodwasp larvae (Erbilgin et al., 2002; Phillips, 2002; Carnegie and Loch, 2010; Yousuf et al., 2014; Wang et al., 2018, 2019). Host tree endophytic fungi such as *Trichoderma harzianum*, *T. viride*, *T. atroviride,* and *Phlebiopsis gigantea* can effectively antagonize *A. areolatum*, reducing its competitive ability compared to other endophytic fungi (Wang et al., 2018). *Ophiostoma minus* and *Aspergillus niger* have been reported to exhibit strong repellent activity against unmated females of *S. noctilio* (Wang et al., 2018). These results investigate the interactions between the native woodwasp and the now sympatric invasive *S. noctilio* based on the signals from fungal volatiles and volatile pheromones in the host location.

*Sirex nitobei* Matsumura is the only native woodwasp that co-habits with the same hosts as *S. noctilio* in some parts of China (Wang et al., 2017). Both wasps attack stressed pines and may compete for habitat. Each wasp carries only one species of symbiotic fungus. *Sirex nitobei* was found to carry either *Amylostereum chailletii* or *A. areolatum* IGS-D (Wang et al., 2021). In China, *S. noctilio* consistently carries *A. areolatum* IGS-BD (Wang et al., 2021). However, when *S. nitobei* and *S. noctilio* shared the same host (Wang et al., 2017), a small proportion of *S. nitobei* individuals carried the invasive *A. areolatum* IGS-BD, a horizontal transmission from *S. noctilio* (Wang et al., 2021). This is because *S. nitobei* acquires the fungus from the host tree, where the symbiotic fungus of *S. noctilio* grows, rather than from the parent during the larval stage (Wang et al., 2021). This suggests that the presence of *A. areolatum* IGS-BD, already established in trees, would not hinder *S. nitobei* from colonizing the same tree. Hence, it is worth exploring whether the established symbiotic fungi of *S. nitobei* in the tree affect the selection behavior of *S. noctilio*. Based on the symbiotic relationship between siricid woodwasps and *Amylostereum* (Tabata et al., 2012; Sarvary et al., 2016), we speculate that the locations of oviposition strongly influence the horizontal acquisition of these symbionts. The objectives of this study were to assess the influence of different genotypes and species of fungi on the behavioral responses of *S. noctilio* in laboratory and field assays during the overlapping flight period. Here, we performed behavioral assays in the laboratory to test the directional movement of female *S. noctilio* and to identify volatile compounds among different genotypes and species of *Amylostereum*. In addition, we examined the oviposition behavior of invasive *S. noctilio* females in pine bolts (sections of wood), inoculated with the aforementioned fungi in the field.

## 2 Materials and methods

### 2.1 Insect rearing

*Sirex noctilio* adults were collected from four forest stands of damaged *Pinus sylvestris* var. *mongolica* in May (before the adult flight period) from Yushu City (YS), Jilin Province. Trees (16–30 cm in diameter at breast height, DBH) were felled, cut into bolts (1 m long), and sent to the quarantine laboratory of the Beijing Forestry University, China. The bolt ends were coated with wax to conserve moisture and to prevent contamination by other fungi, then moved to individual mesh bags at room temperature. Emerged woodwasps were collected daily. They were stored separately in clear plastic cups at 4°C until use. Mating trials were conducted outdoors: woodwasps were put into a square net cage based on a sex ratio of 15:5 (♂: ♀) for observation, and the mated woodwasps were marked for subsequent experiments (Supplementary Figure S1) (Fukuda and Hijii, 1996; Bao et al., 2018). Female insects used for the bioassay were 1–3 days old. The average forewing length was 23.14 ± 5.22 mm.

### 2.2 Fungal culture

Cultures of *Amylostereum* were obtained from mycangia of female adults [according to Thomsen and Harding (2011)]. The arthrospores were then transferred to Petri dishes containing an artificial medium made of potato dextrose agar (PDA) (40 g potato, 4 g glucose, and 3 g agar). Cultures maintained in the dark at ambient temperature (PGX-250A, Beijing, China, 25 ± 1°C) for the duration of each treatment [14 days, studies show that *S. noctilio* is attracted to its volatiles (Martínez et al., 2006; Jofré et al., 2016; Sarvary et al., 2016; Faal et al., 2021)]. Fungal strains were identified according to their morphological features and molecular tools (*A. areolatum* IGS-BD introduced by *S. noctilio*, *A. areolatum* IGS-D, or *A. chailletii* carried by native *S. nitobei*; YS, China) (Thomsen and Harding, 2011; Wang et al., 2021). The source of the odor was 64 cm^2^ of mycelium and culture medium (an amount that elicits a behavioral response) Fernandez Ajo et al., 2015; Sarvary et al., 2016).

### 2.3 Olfactometer assays

*Sirex noctilio* (149 mated and 177 unmated females) were exposed to volatiles in a Y-tube olfactometer (base: 30 cm, arms: 25 cm, diameter: 3 cm, angle between arms: 60°) between 9:00 a.m. and 15:00 p.m. The airflow was 0.1 ± 0.02 L min^−1^ and successively filtered through activated charcoal and distilled water. Petri dishes containing uninoculated PDA stored in the incubator were used as control groups. Two odor sources were enclosed within separate, sealed glass containers. All glassware were thoroughly cleaned using 75% alcohol and distilled water and then dried in an oven at 120°C (Yi Heng, DHG-9140A, Shanghai, China) after repeating each experiment (Wermelinger and Thomsen, 2012). To avoid visual asymmetries in the olfactometer arms and differences in the lighting conditions that could affect woodwasp selection, the arms were switched after each round of five female wasps. If the woodwasp moves to an olfactory arm more than 12.5 cm for at least 10s, it is identified as making a choice. The woodwasp that did not enter an arm after 10 min was considered “No response.” Binomial tests were performed on each responding wasp to assess biases toward fungal volatile vs. clean air.

### 2.4 Wind tunnel and video tracking

**Figure 1:**
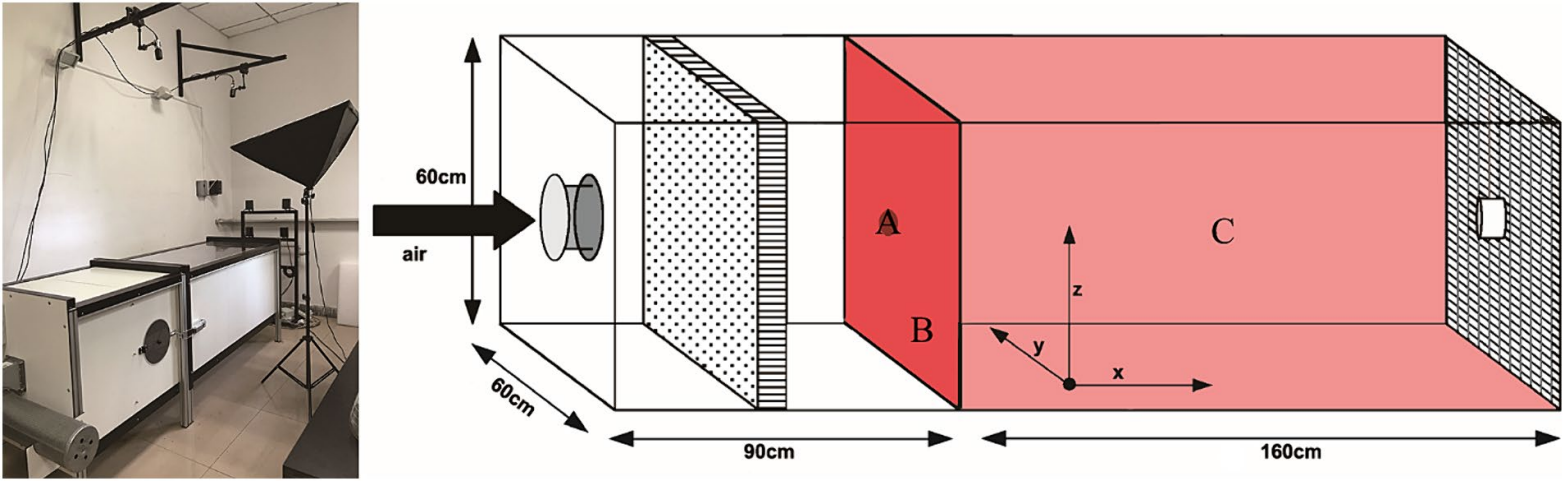
Schematic diagram of wind tunnel. (A) “the source,” (B) “upwind screen,” or (C) “elsewhere” in the arena.

The fungi that attracted females in 2.3 assays have been tested in a wind tunnel to assess their attractiveness at long range under semi-field conditions. Both ends of the wind tunnel are covered with black epoxy metal mesh (160 cm long × 60 cm tall × 60 cm deep, operated at 29 ± 2°C and 70 ± 3% R. H., between 9.00 a.m. and 15.00 p.m.). Petri dishes containing PDA or fungus were used as an odor plume upwind (Figure 1). Woodwasps were released individually from the center of the downwind screen and recorded with two monochrome CCD video cameras (Cohu, San Diego, CA, United States). A software package of EthoVision 3.1, “Track 3D” (Noldus Information Technology, Wageningen, Netherlands), was used for recording 3D track data (30 frames / s). The air velocity was set at 200 mm / s in the positive x-axis (Cooperband et al., 2012; Sarvary et al., 2015). Woodwasps failed to appear on both cameras within 15 min were marked as “No response.” The recording stopped after the woodwasp had been in the camera’s view for more than 10 min. The woodwasp responses were categorized as landing on the upwind screen, the source, or elsewhere in the arena (Figure 1). The odor source (as above mentioned) was changed randomly on different dates.

### 2.5 Ovipositor drilling trials

#### 2.5.1 Fungal inoculation of the experimental bolts

The fungi were cultured in 25°C darkness on PDA for more than 14 days. Circular plugs of different genotype fungi used for inoculating the bolts were cut from the mature cultures based on previous reports (Madden, 1974; Fukuda, 1997). *Pinus sylvestris* var. *mongolica* (with no sign of *Sirex* oviposition) was harvested from a nursery in Jianping County, Liaoning Province. Trees (DBH): 21.97 ± 0.28 cm (Wang et al., 2017) were cut into 100 cm long bolts. Two weeks prior to the experiment, all the bolts were randomly inoculated with the plugs of uninoculated PDA or fungi (*A. areolatum* IGS-BD, *A. areolatum* IGS-D, or *A. chailletii*). A 6-mm diameter electric drill (Lomvum-1201, China) was used to remove 16 “plugs” (approximately 10 mm deep) from the surface of the bolts. Plugs of uninoculated PDA and fungal plugs were placed into holes and then covered with sterilized sawdust (approximately 4 mm thick). There are 16 “plugs” on each bolt, 10 cm apart and 35 cm away from the cross-section of logs, aiming to reduce the potential impact of truncation (Supplementary Figure S2). The unused sawdust is dried in an oven at 80°C for 48 h to weigh and calculate the percentage moisture of the test logs ([% moisture = (wet weight - dry weight) / wet weight × 100]).

#### 2.5.2 Oviposition behavior

The bolts inoculated with fungus or PDA were randomly placed in an outdoor 3 × 3 m mesh tent. Ten *S. noctilio* females were released in each replicate and observed for 8 h each day (between 9:00 a.m. and 17:00 p.m., except for rainy days, for a total of 30 females (15 mated)). We observed and recorded the spawning points and marked them in time by using colored markers. The cylindrical coordinate system was used to evaluate the surface of each bolt, and unique spatial coordinates were assigned to each spawning point on the bolts. This was accomplished by measuring the distance and direction from the closest “fungal plug.”

### 2.6 Volatile sampling and chemical analysis

The volatile organic compounds (VOCs) were collected from 14-day-old *Amylostereum* species (*A. areolatum* IGS-BD, IGS-D, and *A. chailletii*) by headspace solid phase microextraction (HS-SPME) (Wang et al., 2018), using a SPME fiber (65 μm, Supelco, Inc., Bellefonte, PA). The experiment involved three replicates for each strain. The silicon-based compounds present in the volatiles were removed. The preliminary identification of the volatiles was performed by combining the retention index(RI) and the National Institute of Standards and Technology database (NIST). To obtain the relative content of each volatile compound, the peak area was calculated through normalization.

### 2.7 Statistical analysis

One-way analysis of variance (ANOVA) was utilized to compare the response percentages of woodwasps to various odor sources from different fungal species. Tukey’s honestly significant difference (HSD) test was subsequently used to identify statistically significant differences between the fungi. The percentage of *S. noctilio* responding to different target stimuli was analyzed using Pearson’s chi-square test. We used a generalized linear model (GLM), assuming a binomial distribution of residuals, to assess the influence of mating status on selections. Additionally, we included the variable of target stimulus in the analysis, as experiments were conducted with varying target stimuli. The data were fitted to the model: “chosen source = mating status (either mated or unmated) * target stimulus.” All data analyses were performed using IBM SPSS Statistics 26 (Chicago, IL, United States).

Fisher’s exact test was used to assess variations in the percentage of responsive female *S. noctilio* landing on the upwind source, screen, or elsewhere. The track duration of woodwasps in response to different target stimuli was compared using a one-way ANOVA followed by Tukey’s HSD test. Differences in flight parameters (Supplementary Table S1) among treatments were assessed using a GLM, Tukey (T), or Games Howell test, depending on the equality of variances (SPSS 26 for Windows).

In the fungal inoculation experiment, differences in the average distance of spawning points between treatments were evaluated using the non-parametric Kruskal–Wallis H-test followed by Dunn’s test for multiple comparisons. The number of spawning points of woodwasps in response to different target stimuli was compared using a one-way ANOVA followed by Tukey’s HSD test (SPSS 26 for Windows). Log10 is used to convert the minimum distance (cm) for standardizing the data. This calculation was performed in R (version 4.0.3 for Windows). We recorded the distribution of drills in each bolt, treated the drills as spatial points, and estimated the spatial distribution using the maptools and spatstat packages in R (Baddeley et al., 2017). We assess the statistical significance of empirical estimates of k functions for a Monte Carlo simulation of 200 complete spatial randomness (CSR).

One-way ANOVA and Tukey’s HSD tests were performed to examine quantitative differences in concentrations of VOCs emitted by various fungal species. VOCs emitted by different fungi were grouped using principal component analysis (PCA) and cluster analysis. PCA was used to create a plot based on their overall volatile profiles. Hierarchical cluster analysis of each sample was conducted using the between-group linkage method and Euclidean distance. All data were analyzed using IBM SPSS Statistics version 26 for Windows.

## 3 Results

### 3.1 Olfactory responses to compounds of *A. areolatum* IGS-D and *A. chailletii*

**Figure 2:**
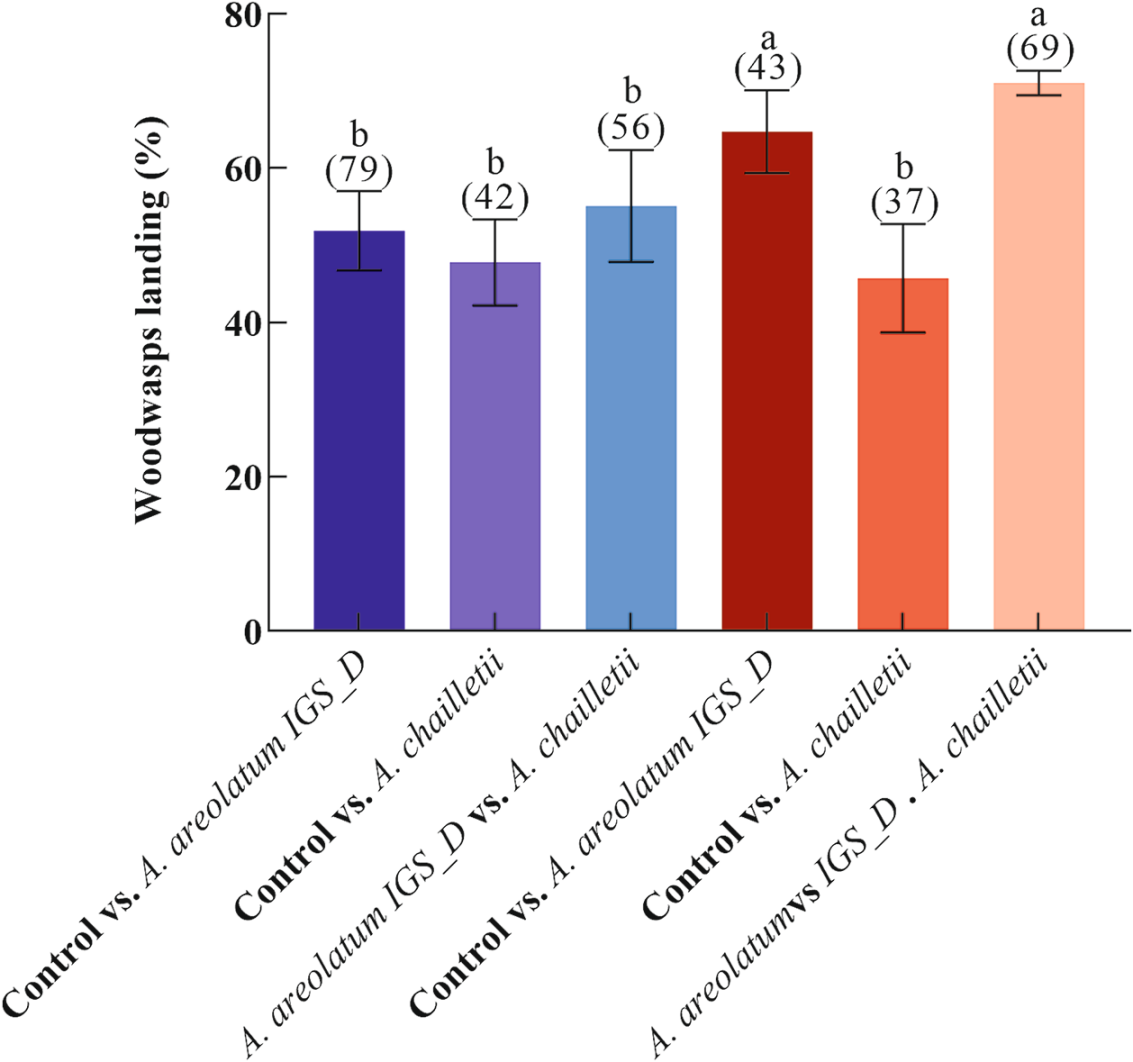
Percentage of *Sirex noctilio* responding to olfactometer stimuli (response) vs. showing no response to any arms (no response). Six independent experiments were conducted using various combinations of odor stimuli, and the percentage of woodwasps responding to these stimuli is presented. Red columns represent mated females, while blue represents unmated females. Numbers in parentheses represent the total number of woodwasps tested in each experiment. Different letters above the bars indicate statistical differences between experiments (*p* < 0.05).

**Figure 3:**
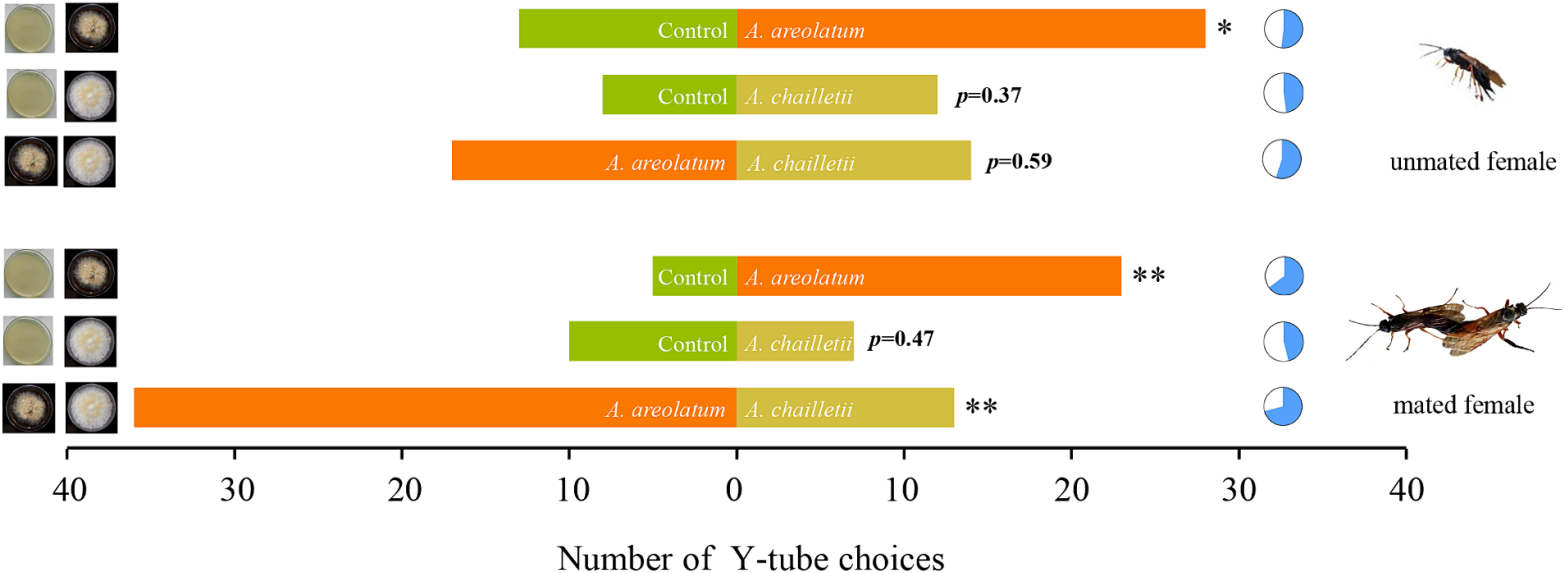
Number of *Sirex noctilio* responding to different treatments. Six independent experiments were conducted for mated and unmated female wasps landing on *Amylostereum areolatum*_D vs. *A. chailletii* cultures. The pie charts indicate the percentage of wasps responding to each treatment and the significant difference by marking with an asterisk (*p*-values are based on Pearson’s chi-square test, ^*^p < 0.05; ^**^p < 0.01).

Varied response levels were observed in female woodwasps to VOCs emitted by different fungi (*F* = 9.301, df = 5, *p* < 0.001; Figure 2). Mated woodwasps exhibited higher response levels when exposed to VOCs emitted by *A. areolatum* IGS-D and *A. chailletii* (65–71%). The lowest response level was observed in mated woodwasps exposed to the control group and *A. chailletii* (46%). Comparisons of the choices made by the responding wasps showed no bias toward any of the olfactometer arms when clean air was presented (*p* > 0.05). As shown in Figure 3, virgin females were attracted to *A. areolatum* IGS-D (χ^2^ = 5.488, *p* < 0.05) and *A. chailletii* (χ^2^ = 0.8, *p* = 0.37). There were no statistically significant differences in the proportion of unmated wasps that responded to the target stimulus arms (*A. areolatum* IGS-D vs. *A. chailletii*; χ^2^ = 0.29, *p* = 0.59). Mated females of *S. noctilio* displayed a strong response to the 14-day-old fungal culture; they responded positively to *A. areolatum* IGS-D (χ^2^ = 10.796, *p* < 0.001) (Figure 3). The target stimulus had the greater impact on the chosen source (GLM, χ^2^ = 29.816, *p* < 0.001), particularly evident for mated females (GLM, χ^2^ = 8.675, *p* < 0.05).

**Figure 4:**
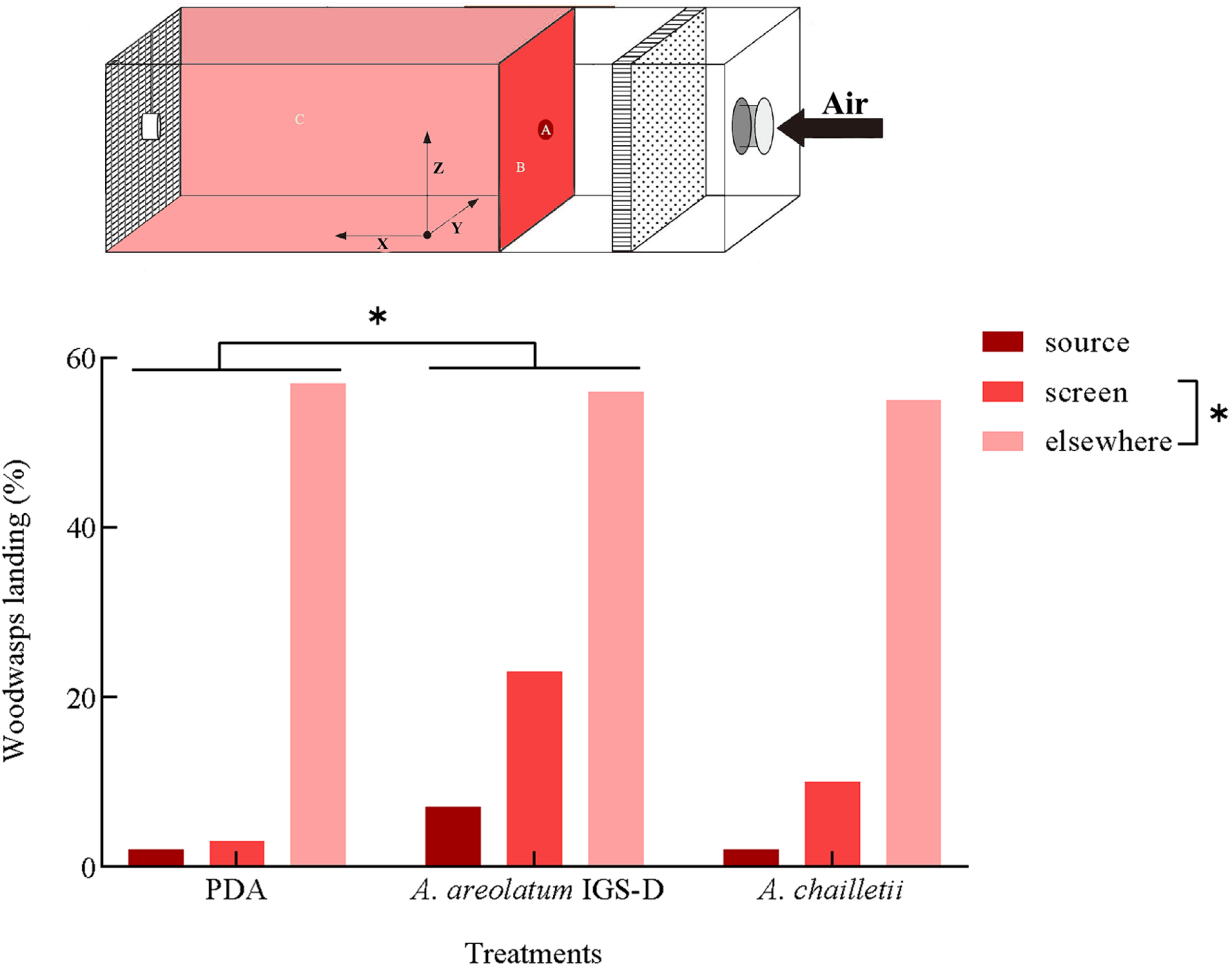
Percentage of responding *Sirex noctilio* females landing on the upwind source, screen, or elsewhere in the arena per treatment. Percentage (%) = percentage of woodwasps leaving the release site within 5 min (Pearson’s chi-square test, *p* < 0.05).

**Figure 5:**
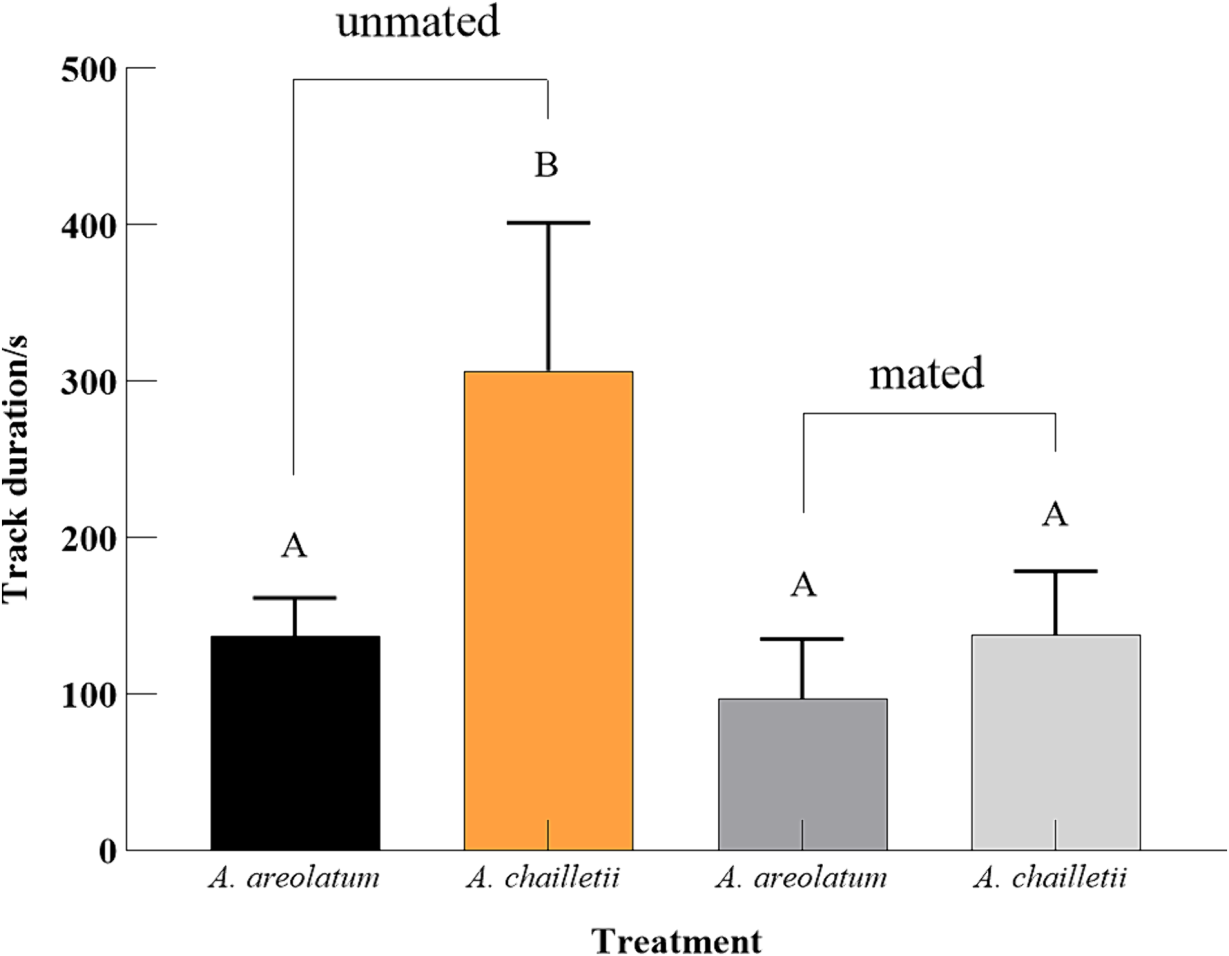
Mean track duration with four different treatments. Responding woodwasps landed on the upwind screen (one-way ANOVA followed by Tukey’s HSD test, *p* < 0.001).

Out of the 235 woodwasps tested, 215 (90%) responded by flying upwind. The upwind flight response differed significantly between treatments (χ^2^ = 11.388, *p* < 0.05). The distribution of landing areas exhibited statistically significant variation when exposed to *A. areolatum* IGS-D vs. PDA (χ^2^ = 9.519, *p* < 0.05). When exposed to the *A. areolatum* IGS-D, the percentage of landings on the upwind screen reached up to 23% (Figure 4: screen, *n* = 94). Volatiles from *A. areolatum* IGS-D caused 7% of the woodwasps that responded to land on the source center, a phenomenon not observed in other treatments (Figure 4: source). The track duration was greater for virgin females that landed on *A. chailletii* (Figure 5; *t* = 306.9 s, *F* = 13.01, *p* < 0.01) than for those exposed to *A. areolatum* IGS-D. The trajectories were reconstructed into 3D images, as shown in Supplementary Figure S3. The trajectories revealed that woodwasps tended to fly upwind along the x-axis in all treatments. The average flight speed exposed to *A. areolatum* IGS-D volatiles was 88.06 ± 19.94 mm/s. Woodwasps were more rapidly activated in the presence of a plume of *A. areolatum* IGS-D compared to those exposed to PDA (Table 1) (GLM, *p* < 0.05). Woodwasps exposed to *A. areolatum* IGS-D and *A. chailletii* exhibited significant differences in Path 3D and Path x-z (Path 3D: GLM, p < 0.05; Path x-z: GLM, p < 0.05). No statistical differences were observed among the other flight parameters (Table 1, *p* > 0.05).

**Table 1:** Summary of parameter values (mean ± SE) obtained from 3D flight tracks of *Sirex noctilio* responding to three treatments: uninoculated PDA (ck), *Amylostereum areolatum* IGS-D, and *A. chailletii*.

Parameter		Treatment means	
	ck	*Amylostereum areolatum* IGS-D	*Amylostereum chailletii*
Activation, s	101.3 ± 16.80 (*n* = 10) a^b^	46.48 ± 8.304 (*n* = 50) b^b^	63.50 ± 20.03 (*n* = 14) ab^b^
Flight speed 3-D, mm s^−1^	53.80 ± 21.78 (*n* = 9)	88.06 ± 19.94 (*n* = 17)	42.97 ± 7.196 (*n* = 13)
Path 3D, mm	4,362 ± 1,366 (*n* = 9) ab^a^	7,680 ± 1,310 (*n* = 17) a^a^	3,795 ± 554.3 (*n* = 13) b^a^
Path x–y, mm	3,288 ± 1,104 (*n* = 9)	4,681 ± 667.5 (*n* = 17)	2,828 ± 341.2 (*n* = 13)
Path x–z, mm	3,620 ± 1,061 (*n* = 9) ab^b^	6,633 ± 1,223 (*n* = 17) a^b^	3,202 ± 498.5 (*n* = 13) b^b^
Tortuosity 3D	3.40 ± 0.67 (*n* = 9)	4.50 ± 0.89 (*n* = 17)	2.67 ± 0.41 (*n* = 13)
Angular velocity 3-D, ° s^−1^	857 ± 72.2 (*n* = 9)	933.5 ± 35.69 (*n* = 17)	925.6 ± 38.79 (*n* = 13)
Angular change 3-D	28.58 ± 2.41 (*n* = 9)	31.39 ± 1.09 (*n* = 17)	32.14 ± 0.85 (*n* = 13)
track xv	63.08 ± 7.28 (*n* = 9)	76.85 ± 1.82 (*n* = 17)	65.28 ± 3.79 (*n* = 13)
course xv	9.70 ± 2.23 (*n* = 9)	10.38 ± 1.51 (*n* = 17)	9.56 ± 2.64 (*n* = 13)

Treatment means were calculated from the means of measurements for each frame in replicates captured at a rate of 30 frames per second. Different letters within a column indicate significant differences between treatments (GLM, *p* < 0.05). n = Number of included flights. SE, standard error.

a Equal variances, Tukey (T).

b Unequal variances, Games Howell.

### 3.2 Symbiont-mediated oviposition choice

**Figure 6:**
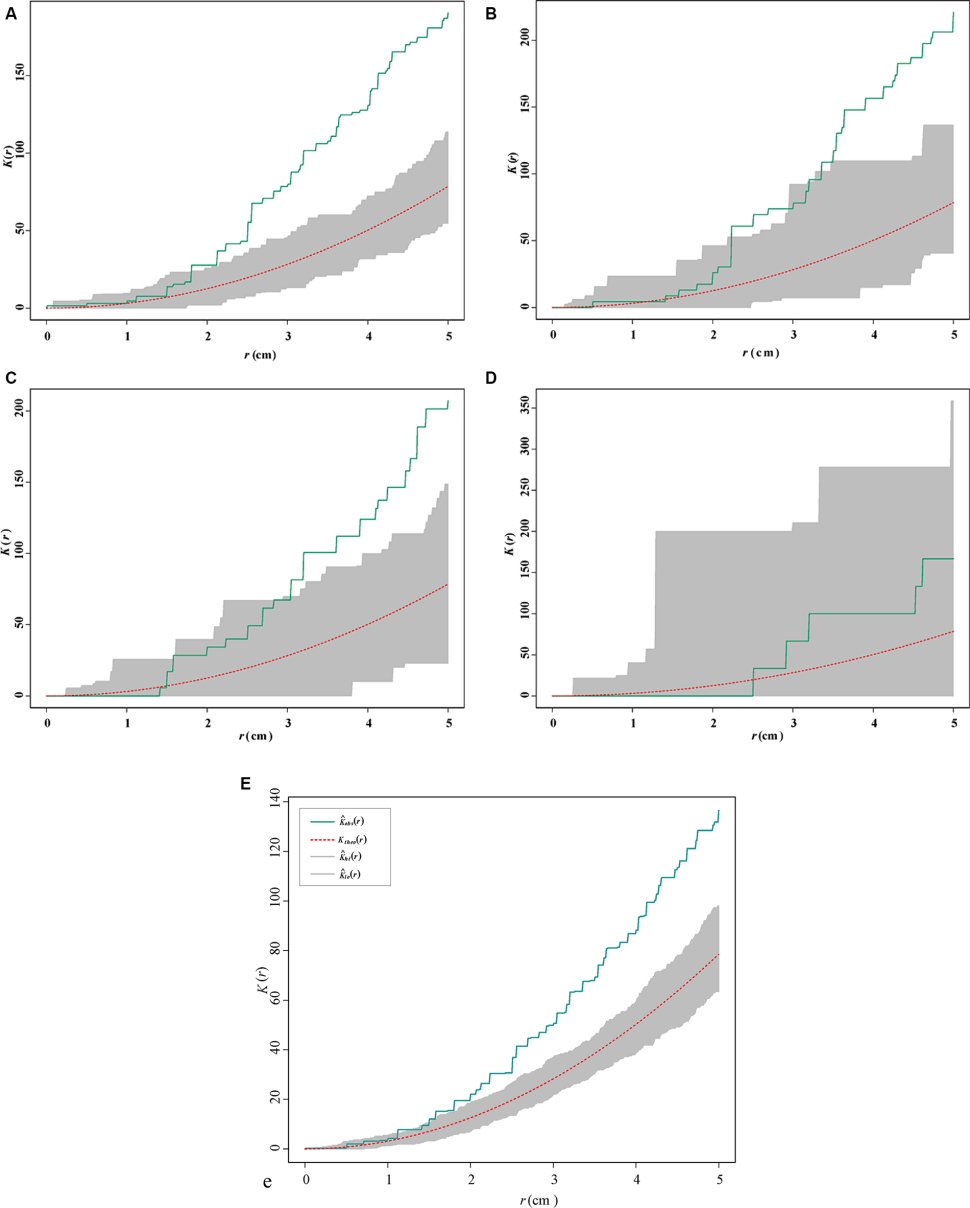
Ripley *K*-function estimates (solid green line) of the spatial location of the spawning points for *Sirex noctilio* in 2019 **(A)** *Amylostereum areolatum*_BD, **(B)** *A. areolatum*_D, **(C)** *A. chailletii*, **(D)** ck (uninoculated PDA), and **(E)** all treatments. The red dotted lines indicate the expected values under complete spatial randomness (CSR) based on 200 simulations.

The origin of the coordinate represents the inoculation points, and the points of different colors represent the spawning points of *S. noctilio* females under different treatments (Supplementary Figure S4). All the bolts had moisture levels ranging from 43.3 to 47.5%, indicating a medium moisture content environment (Madden, 1968; Hajek et al., 2018). Inoculated fungi were isolated from most of the bolts. We observed significant spatial aggregation in the location of the spawning points when the spatial scale >3.5 cm (Figure 6), except for the control treatment. The *K*-function estimates of fungal-treated bolts were similar but different from those of the control (CSR) (Figure 6).

**Figure 7:**
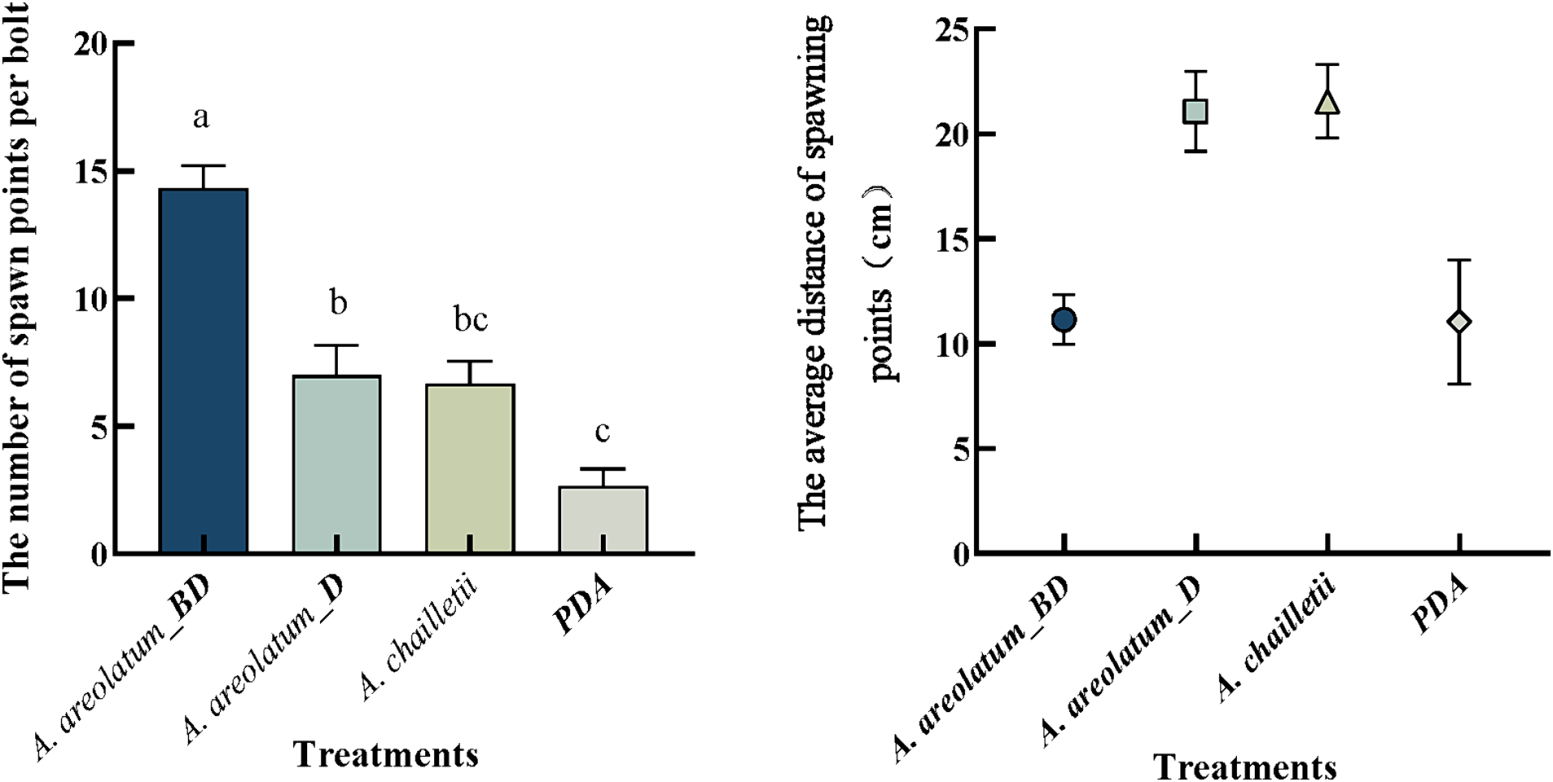
Number of spawning points per bolt and the average distance between spawning points and control or fungal plus. Different uppercase letters represent significant differences among the treatments (*p* < 0.05).

In terms of spawning points per bolt, we found significant differences among treatments (Figure 7). Selection tests displayed that *S. noctilio* drilled more into bolts with the invasive *A. areolatum* IGS-BD (*n* = 43, *p* < 0.05) and the least drilling into control bolts (*n* = 8). Mean distances between spawning points and “fungal plus” or control were presented in Figure 7. We observed a significant treatment effect (*p* < 0.001). *Sirex noctilio* females drilled closest to *A. areolatum* IGS-BD (11.15 ± 1.19 cm) and PDA, and furthest to *A. chailletii* (21.55 ± 1.75 cm).

### 3.3 Composition of the fungal volatiles

**Figure 8:**
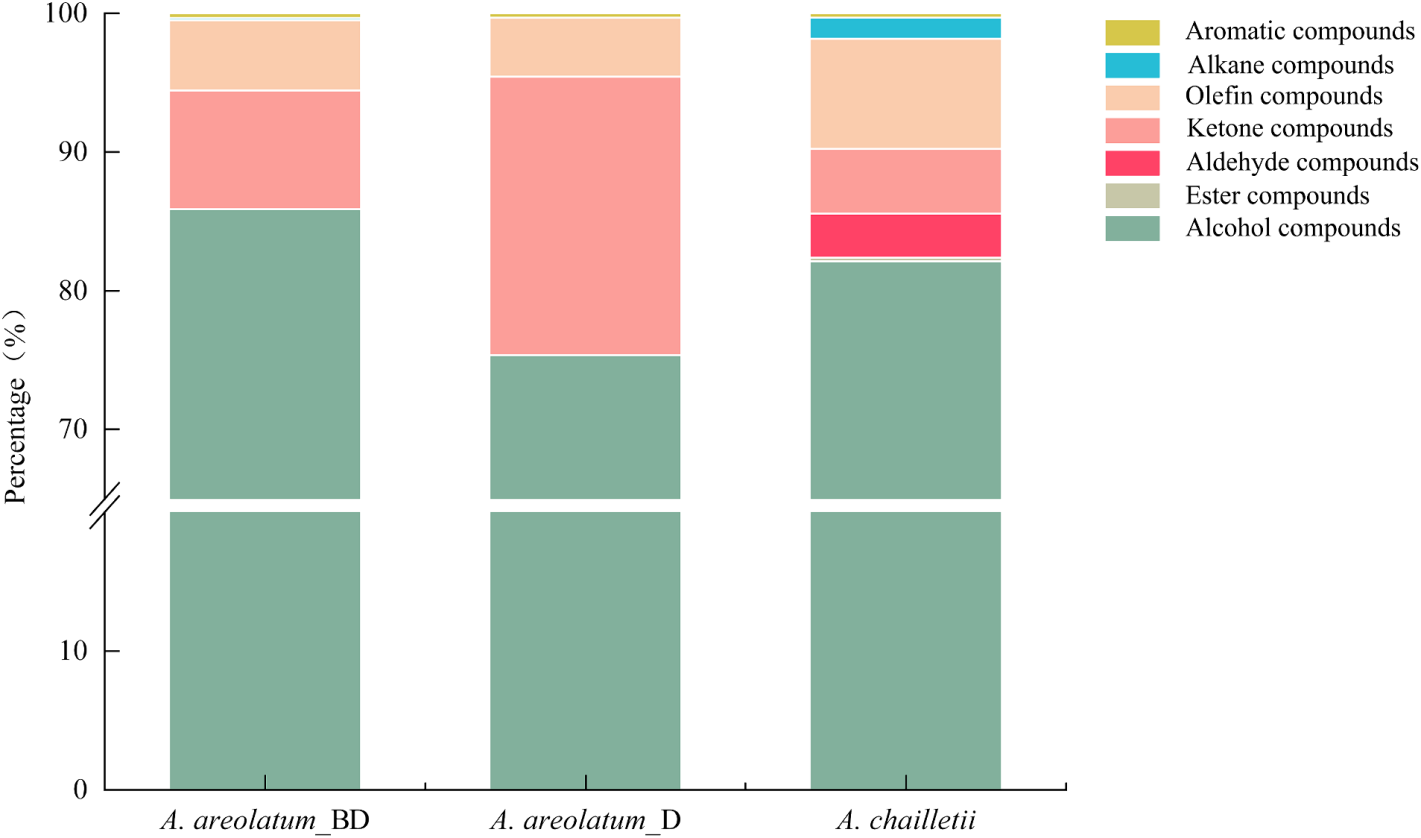
Relative content of volatile compound groups of *Amylostereum areolatum* (IGS_BD, D) and *A. chailletii.*

The basic chemical composition of *A. areolatum* (IGS-BD or D) and *A. chailletii* were different. Forty-one VOCs were identified in fungal samples grown for 14 days. The major component of fungi volatiles was 1-octen-3-ol (59.06% ~ 71.93%) (Supplementary Figure S5; Table 2). The extracted volatiles mainly include alcohol (75.34% ~ 85.87%), aldehyde, ketone, and terpene (Figure 8).

**Table 2:** Comparison of main volatile components between *Amylostereum areolatum*_BD, *A. areolatum*_D, and *A. chailletii.*

No.	CAS	Compound	Content %			*F*-value
			*A. areolatum*_BD	*A. areolatum*_D	*A. chailletii*	
1	71–41-0	1-Pentanol	/	0.04 ± 0.04	0.01 ± 0.01	–
2	592–43-8	2-Hexene	3.83 ± 0.29^b^	3.36 ± 0.09^b^	6.56 ± 0.22^a^	50.80^*^
3	4,312-99-6	1-Octen-3-one	0.7 ± 0.19^b^	0.63 ± 0.16^b^	1.48 ± 0.11^a^	9.74^*^
4	3,391-86-4	1-Octen-3-ol	71.93 ± 2.04^a^	59.06 ± 2.16^b^	68.53 ± 0.4^a^	15.10^*^
5	106–68-3	3-Octanone	8.51 ± 2.13^b^	19.47 ± 2.25^a^	3.11 ± 0.43^c^	23.19^*^
6	767–12-4	Cyclohexanol, 3,3-dimethyl-	0.44 ± 0.25	/	/	–
7	589–98-0	3-Octanol	0.47 ± 0.26^ab^	0.96 ± 0.33^a^	/^b^	5.37^*^
8	22,104–78-5	2-Octen-1-ol	5.55 ± 0.37	5.66 ± 0.19	6.72 ± 0.71	–
9	5,709-98-8	3-Cyclohexene	0.01 ± 0.01^b^	0.37 ± 0.19^b^	0.95 ± 0.13^a^	19.82^*^
10	592–57-4	1,3-Cyclohexadiene	0.21 ± 0.16	0.02 ± 0.02	/	–
11	111–87-5	1-Octanol	3.67 ± 0.27^b^	3.47 ± 0.21^b^	5.3 ± 0.28^a^	13.82^*^
12	25,246–27-9	Alloaromadendrene	0.2 ± 0.03^b^	0.24 ± 0.06^ab^	0.38 ± 0.05^a^	4.33^*^
13		trans-Sesquisabinene hydrate	0.07 ± 0.05	/	/	–
14	1,461-03-6	1H-Benzocycloheptene, 2,4a,5,6,7,8-hexahydro-3,5,5,9-tetramethyl-, (R)-	/	0.05 ± 0.02	/	–
15	644–30-4	Benzene, 1-(1,5-dimethyl-4-hexenyl)-4-methyl-	0.16 ± 0.1	0.14 ± 0.04	/	–
16	110–83-8	Cyclohexene	0.5 ± 0.09^a^	0.12 ± 0.02^b^	0.06 ± 0.02^b^	16.55^*^
17	78–70-6	Linalool	0.3 ± 0.16^b^	1.92 ± 0.21^a^	/^b^	56.68^*^
18	128–37-0	Butylated Hydroxytoluene	0.11 ± 0.07	/	/	-
19	489–41-8	(−)-Globulol	0.6 ± 0.33^ab^	1.25 ± 0.18^a^	0.01 ± 0.01^b^	7.24^*^
20	17,699–05-7	Bicyclo[3.1.1]hept-2-ene, 2,6-dimethyl-6-(4-methyl-3-pentenyl)-	0.07 ± 0.07	0.05 ± 0.03	/	–
21	54,789–22-9	1H-Inden-1-one, 2,3-dihydro-3,3,5,6-tetramethyl-	/^b^	0.12 ± 0.02^a^	/^b^	133.35^*^
22	97,452–08-9	3-Ethyl-4,4-dimethyl-2-(2-methylpropenyl) cyclohex-2-enone	0.03 ± 0.02	/	/	–
23	88,919–66-8	Cyclobutane, tetrakis(1-methylethylidene)-	0.12 ± 0.05^a^	/ ^b^	/ ^b^	5.74^*^
24	15,352–77-9	beta-bisabolol	2.45 ± 1.46	2.85 ± 0.66	/	–
25	515–69-5	alpha-Bisabolol	0.03 ± 0.03	0.03 ± 0.01	/	–
26	37,841–91-1	Cycloprop[e]indene-1a,2(1H)-dicarboxaldehyde	0.03 ± 0.02^b^	/^b^	0.33 ± 0.11^a^	5.46^*^
27	3,242-08-8	Cyclohexane, 1-ethenyl-1-methyl-2-(1-methylethenyl)-4-(1-methylethylidene)-	0.03 ± 0.02	/	/	–
28	123,278–27-3	1,3-Cyclopentadiene, 1,3-bis(1-methylethyl)-	/ ^b^	0.09 ± 0.05^a^	/^b^	6.25^*^
29	87,745–32-2	Glaucyl alcohol	/^b^	0.1 ± 0.05^a^	/^b^	8.21^*^
30	110–62-3	Pentanal	/^b^	/^b^	0.13 ± 0.02^a^	27.93^*^
31	4,798-44-1	1-Hexen-3-ol	/ ^b^	/^b^	0.02 ± 0.01^a^	5.39^*^
32	107–86-8	2-Butenal, 2-methyl-	/^b^	/^b^	0.07 ± 0.01^a^	69.28^*^
33	111–27-3	1-Hexanol	/^b^	/^b^	0.21 ± 0.05^a^	10.99^*^
34	103–11-7	2-Ethylhexyl acrylate	/	/	0.15 ± 0.07	–
35	16,747–50-5	Cyclopentane, 1-ethyl-1-methyl-	/^b^	/^b^	1.5 ± 0.3^a^	15.81^*^
36	63,922–44-1	3-Heptyne-2,6-dione, 5-methyl-5-(1-methylethyl)-	/^b^	/^b^	0.09 ± 0.01^a^	23.69^*^
37	2,548-87-0	2-Octenal, (E)-	/ ^b^	/ ^b^	3 ± 0.79^a^	8.99^*^
38	18,937–66-1	Azulen-2-ol, 1,4-dimethyl-7-(1-methylethyl)-	/^b^	/^b^	0.03 ± 0.02^a^	–
39	5,508-58-7	Andrographolide	/^b^	/^b^	0.11 ± 0.03^a^	7.49^*^
40	35,192–73-5	1-Nonen-4-ol	/ ^b^	/ ^b^	0.8 ± 0.29^a^	4.83^*^
41	105–31-7	1-Hexyn-3-ol	/ ^b^	/ ^b^	0.49 ± 0.39^a^	–

/ indicate not detected, means ± standard error. ^a,b,c^ Lowercase superscripts indicate statistically significant differences (one-way ANOVA followed by Tukey’s HSD test, *p* < 0.05).

**Figure 9:**
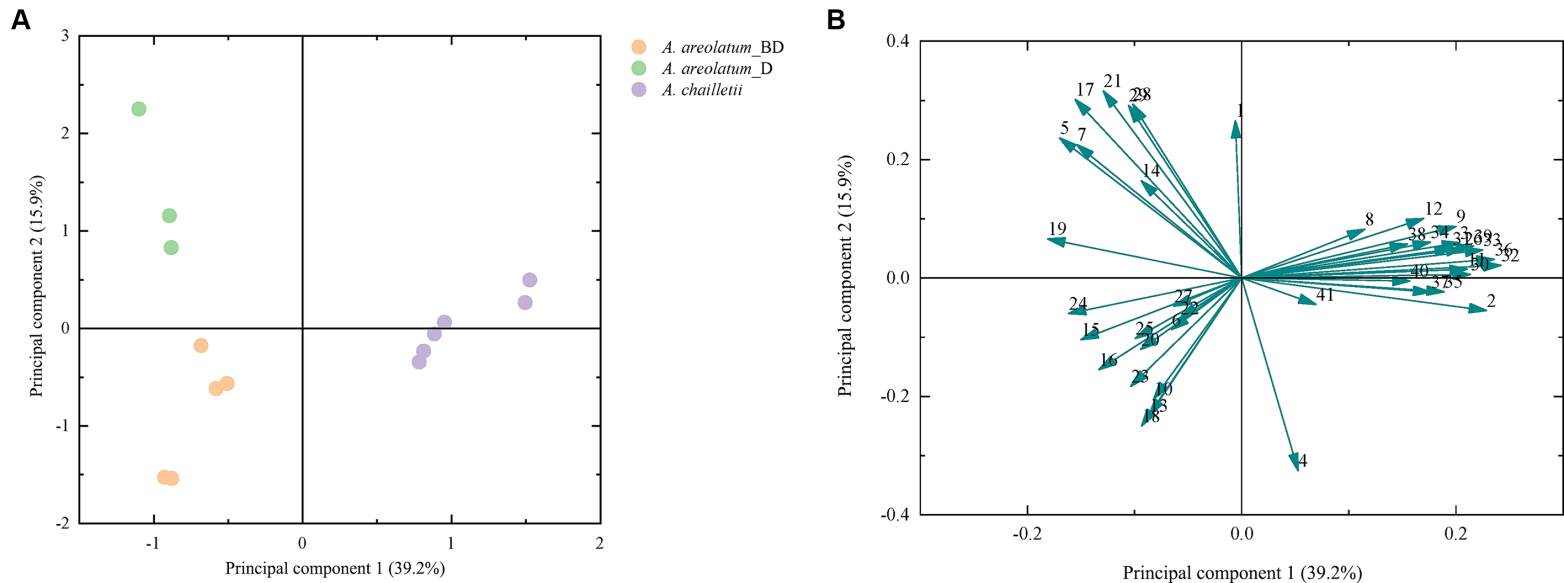
Score plot **(A)** and loading plot **(B)** from PCA of volatile compounds of *Amylostereum areolatum* (IGS_BD, D) and *A. chailletii*. Each number corresponds to Table 2.

**Figure 10:**
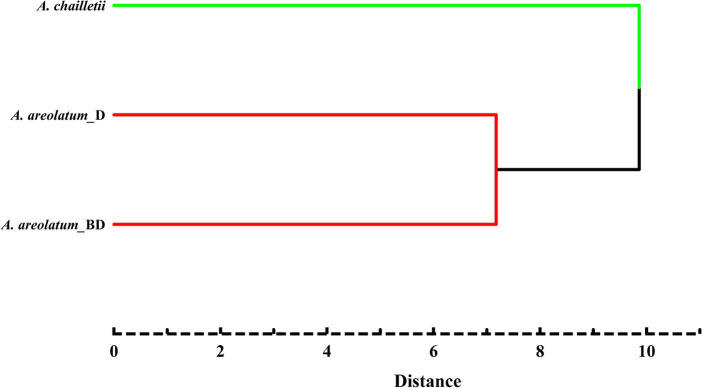
Cluster analysis diagram of volatiles of *Amylostereum areolatum* (IGS_BD, D) and *A. chailletii* based on Euclidean distance.

Figure 9 shows the PCA score plot (A) and loading plot (B) of volatile compounds. The PCs explained 39.2 and 15.9% of the variance (PC1 and PC2, respectively) (Figure 9). PC1 was strongly influenced by 2-butenal,2-methyl -, 3-heptyne-2,6-dione,5-methyl-5-(1-methylethyl)-, 2-hexene and 1-hexanol (2, 32, 33, 36) on the positive axis. As for PC2 on the positive side, 1H-Inden-1-one, 2,3-dihydro-3,3,5,6-tetramethyl-, linalool, 1,3-cyclopentadiene, 1,3-bis(1-methyl)- and glaucyl alcohol (17, 21, 28, 29) showed high loading (Figure 9B). Based on the hierarchical cluster analysis between-groups linkage (squared Euclidean distance), at a distance >7.2 and < 9.9, the samples were clustered into two groups. *Amylostereum areolatum* IGS-BD and D were grouped into one group, which means that the samples share similar characteristics. The main difference between the two clusters is that there are aldehydes and alkanes in the volatile compounds of *A. chailletii* (Figure 10).

## 4 Discussion

### 4.1 Behavioral responses of *Sirex noctilio* to the symbiotic fungi that are in association with *S. nitobei*

*Sirex noctilio* females were attracted to *Amylostereum areolatum* IGS-D and *A. chailletii*, and mated females were significantly more active. This phenomenon is similar to that observed in South America and North America (Fernandez Ajo et al., 2015; Sarvary et al., 2015). It was also shown that there were significant differences in flight behavior between treatment groups during long-range directional upwind flight, especially in the odor source region. The results suggest that the overall response level is enhanced by fungal volatiles, implying that a heavily infested tree emits substantial amounts of attractive material. *Sirex noctilio* females drilled most into bolts inoculated with *A. areolatum* IGS-BD rather than into control bolts (Madden, 1974; Fernandez Ajo et al., 2015). Similarities between the volatile profiles of *A. areolatum* and those of Eurasian origin may be due to innate responses (Madden, 1988; Sarvary et al., 2016). Drillings in bolts inoculated with symbiotic fungi of *S. nitobei* were at an intermediate level. Consistent with these findings, females can detect *A. areolatum* volatiles in a semi-field setting (Fernandez Ajo et al., 2015; Sarvary et al., 2016; Wang et al., 2018).

It was observed that *S. noctilio* drilled the furthest distance (21.55 ± 1.75 cm) toward *A. chailletii*. Wooding et al. (2013) reported that very few *S. noctilio* individuals can carry *A. chailletii.* This phenomenon has not been found in China (Wang et al., 2021). *Amylostereum areolatum* and *A. chailletii* were only somatically incompatible, which is consistent with past results (Hajek et al., 2013; Castrillo et al., 2015). *Amylostereum chailletii* did not completely inhibit different genotypes of *A. areolatum*. Therefore, that may be a strategy to improve *S. noctilio* adaptability and potentially reduce the competitive antagonism with *A. chailletii* being pre-inoculated (Margrete Thomsen and Koch, 1999; Vasiliauskas and Stenlid, 1999).

A relatively uniform distribution of spawning sites of *S. noctilio* in *Pinus radiata* was noted by Madden (1974) in Tasmania. However, drilling was clustered in more resistant trees (Madden, 1974). This occurs because *S. noctilio* injects symbiotic fungi and toxins during oviposition, creating a conducive microenvironment for offspring development (Slippers et al., 2012). However, drill aggregation enhances the delivery of toxins and fungi, which synergistically resist tree defenses. Our findings are consistent with the phenomenon of *S. noctilio* in North America and the Southern Hemisphere making more drills to overcome tree defenses (Coutts and Dolezal, 1969; Madden, 1974; Thompson et al., 2014; Hajek et al., 2018). The drills of the control group were relatively random, suggesting that the woodwasp chose to inject venom and symbiotic fungi into the host to pre-condition wood (“pre-infection”) (Madden, 1974; Xu et al., 2019a).

Hajek et al. (2018) observed that females preferred wood without prior fungal emplacement (*A. areolatum* IGS-BD carried by *S. noctilio* or *A. areolatum* IGS*-*BE carried by *S. nitidus*) inconsistent with the report of attraction to fungal volatiles (*A. areolatum* IGS*-*BD) and our results, it is possible that the effects observed in those studies are mediated by chemicals operating at different distance scales (Sarvary et al., 2016; Hajek et al., 2018). Studies have shown that both the species and physiological conditions of host trees can affect the drills’ distribution (Hajek et al., 2018; Xu et al., 2019a). It has been hypothesized that differences in host species (North America: *P. sylvestris* or *P. resinosa*), invasion duration, and habitat in newly colonized areas may ultimately lead to different oviposition patterns. (Madden, 1974; Hoebeke et al., 2005; Hajek et al., 2018; Xu et al., 2019a).

### 4.2 The influence of symbiotic fungi volatiles on *Sirex noctilio*’s behavior and biological control

A total of 41 volatile components were identified by SPME-GC–MS, of which alcohol compounds were the most common. Some VOCs differ from those previously reported, possibly due to differences in the method and duration of volatile extraction (Bryant, 2010; Jofré et al., 2016; Sarvary et al., 2016; Faal et al., 2021; Masagué et al., 2023). Previous studies suggest that 2-hexene (6.56 ± 0.22%), Cycloprop[e] indene1a, 2(1H)-dicarboxaldehyde, and (−) -Globulol emitted by *A. chailletii* were attractant compounds (Wang et al., 2018). These compounds with a molecular weight of approximately 200 demonstrate sufficient volatility to act as airborne attractants at long distances (Nation, 2008; Wang et al., 2018). However, some alkane compounds and aldehyde compounds unique to *A. chailletii* may contain antifungal substances, which lead to the antagonistic effect on *A. areolatum*. Earlier findings revealed that *A. chailletii* had an obvious repelling effect on *A. areolatum*, but did not kill it (Hajek et al., 2013; Castrillo et al., 2015). This may also be the reason why *S. noctilio* drills farthest from *A. chailletii*.

The successful invasion of *Dendroctonus valens* (its associated fungus enhances the production and release of 3-carene) and *Bursaphelenchus xylophilus* (using chemicals already present at the invasion area) showed the role of semiochemicals in enhancing the biological invasion (Yan et al., 2005; Zhao, 2006; Lu et al., 2010). The volatiles of native *A. areolatum* IGS-D were similar to those of invasive *A. areolatum* IGS-BD, both of which could attract *S. noctilio* drilling aggregate. It may be more advantageous for females to quickly find a host tree that has been inoculated with symbiotic fungi that provide nutrients to their offspring.

It was speculated that the synergistic effect of the volatiles of native symbiotic fungi and host trees would be conducive to the colonization of *S. noctilio*. According to the bioassay results of our study, the volatiles of the symbiotic fungi can be mixed to prepare an effective attractant. The lures may be adjusted by optimizing the release rates of attractive different genotypes of *A. areolatum* fungal VOCs in the future (Faal et al., 2021).

The natural enemy, *Ibalia leucospoides* (“double-edged sword”) can locate the wasp larvae by symbiotic fungus VOCs (Madden, 1968; Spradbery, 1970; Martínez et al., 2006; Bryant, 2010; Pietrantuono et al., 2012; Robertson, 2014; Jofré et al., 2016; Faal et al., 2021). It was speculated that *S. noctilio* carrying different symbiotic fungi in this study can be a reliable signal for native parasitoids (*I. leucospoides* (Hochenw) (Wang, 2017)) as linalool can attract *I. leucospoides* females (Faal et al., 2021). However, linalool was not detected in *A. chailletii,* which could protect its host, *S. nitobei* (which carries *A. chailletii*), to a certain extent. In northeastern China, *Sirex noctilio* has often been present at low population levels, making it difficult to obtain individuals to conduct further field experiments. We suggest that further studies can be carried out to investigate the differences in volatile compounds between hosts after inoculation with different genotypes of symbiotic fungi and to assess their attractiveness to both woodwasps and their parasitoids in laboratory and field assays.

## 5 Conclusion

The current findings lead us to assume that females of *S. noctilio* can detect trees previously attacked by *S. nitobei* with fungal volatiles. Different genotypes of *A. areolatum* (IGS-BD and IGS-D) and *A. chailletii* were able to detect these compounds, which are attractive to *S. noctilio*, 2-hexene (6.56 ± 0.22%), cycloprop[e] indene-1a, 2 (1H)-dicarboxaldehyde, and (−) -globulol. *Sirex noctilio* is preferred to its symbiotic fungi (*A. areolatum* IGS-BD) but is furthest from *A. chailletii*. That may be a strategy to increase adult fitness and lessen the volatile-mediated antagonistic effects of *A. chailletii*. Studying the response of female woodwasps to these native fungi will help to understand the spatial distribution of woodwasps. Different species of siricids can co-infest the same trees; accompanied by fungal horizontal transmission, which could make pest management more difficult. The role of fungal volatiles may provide improved tools for surveying and dynamic monitoring of pests.

## Data availability statement

The original contributions presented in the study are included in the article/Supplementary material, further inquiries can be directed to the corresponding authors.

## Ethics statement

The manuscript presents research on animals that do not require ethical approval for their study.

## Author contributions

MW: Data curation, Formal analysis, Methodology, Resources, Writing – original draft, Writing – review & editing. CG: Resources, Writing – review & editing. QX: Resources, Writing – review & editing. NF: Resources, Writing – review & editing. JL: Resources, Writing – review & editing. LR: Conceptualization, Funding acquisition, Methodology, Project administration, Supervision, Writing – review & editing. YL: Conceptualization, Funding acquisition, Methodology, Project administration, Supervision, Writing – review & editing.
