# Supplementary material for: Different genotypes and species of symbiotic fungi mediate the behavioral response of invasive Sirex noctilio fabricius (Hymenoptera: Siricidae)
Source: Front Microbiol. 2024 Jul 11;15:1341646. doi: 10.3389/fmicb.2024.1341646 (PMC11269189; doi:10.3389/fmicb.2024.1341646)
Supplement: Supplementary file 1 [file Table_1.DOCX]

Supplementary Material

# Supplementary Figures and Tables

## Supplementary Figures


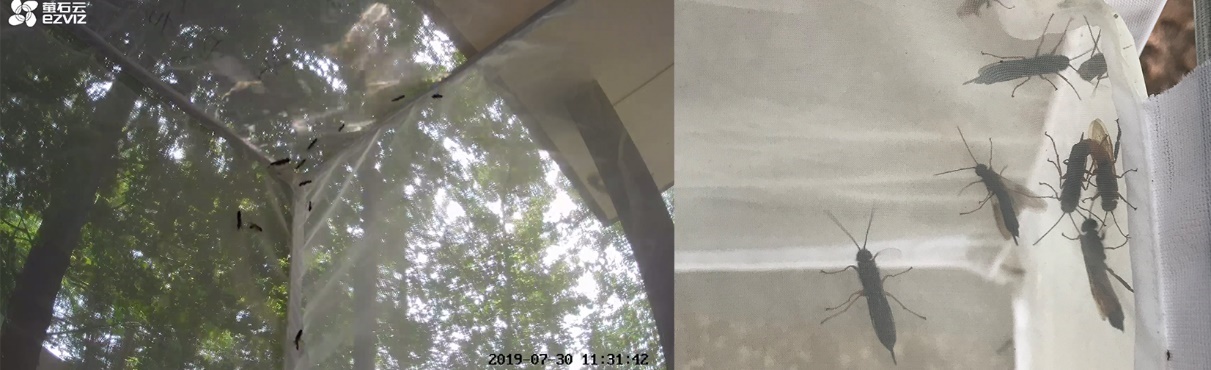


**Supplementary Figure 1** **Mating behavior of *Sirex noctilio***


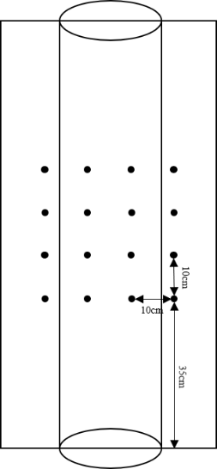


**Supplementary Figure 2 Location and distribution of holes along the tree stem for fungus inoculation. The black dots indicate the inoculation point of fungal treatments or controls around the bolt**


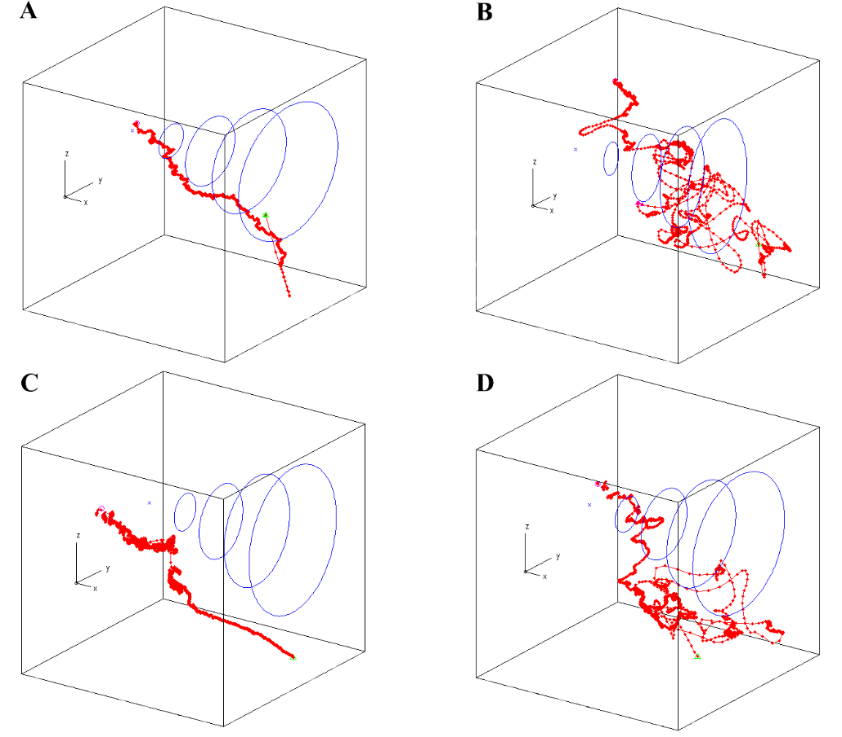


**Supplementary Figure 3**  **Selected 3-D tracks of the upwind flight of *Sirex noctilio* females in the wind tunnel in response to each of the four treatments: (A) unmated female - *A. areolatum_*D cultures; (B) mated female - *A. areolatum_*D cultures; (C) unmated female -** ***A. chailletii* cultures; (D) mated female - *A. chailletii* cultures**


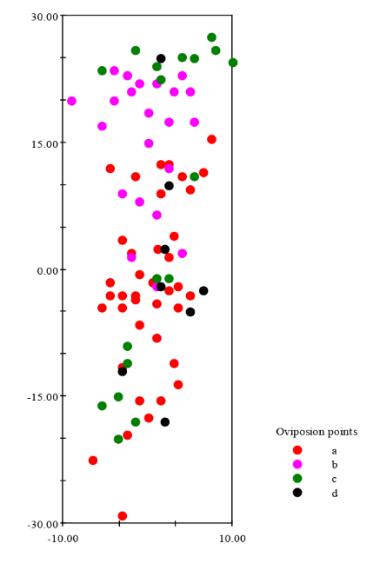


**Supplementary Figure 4 The distribution of oviposition points by *Sirex noctilio* on the bolts. The four treatments were inoculated separately: (a) *Amylostereum areolatum*_BD (introduced with *S. noctilio*); (b) *A. areolatum*_D (carried by *S. nitobei*); (c) *A. chailletii* (carried by *S. nitobei*); (d) ck (uninoculated PDA).**


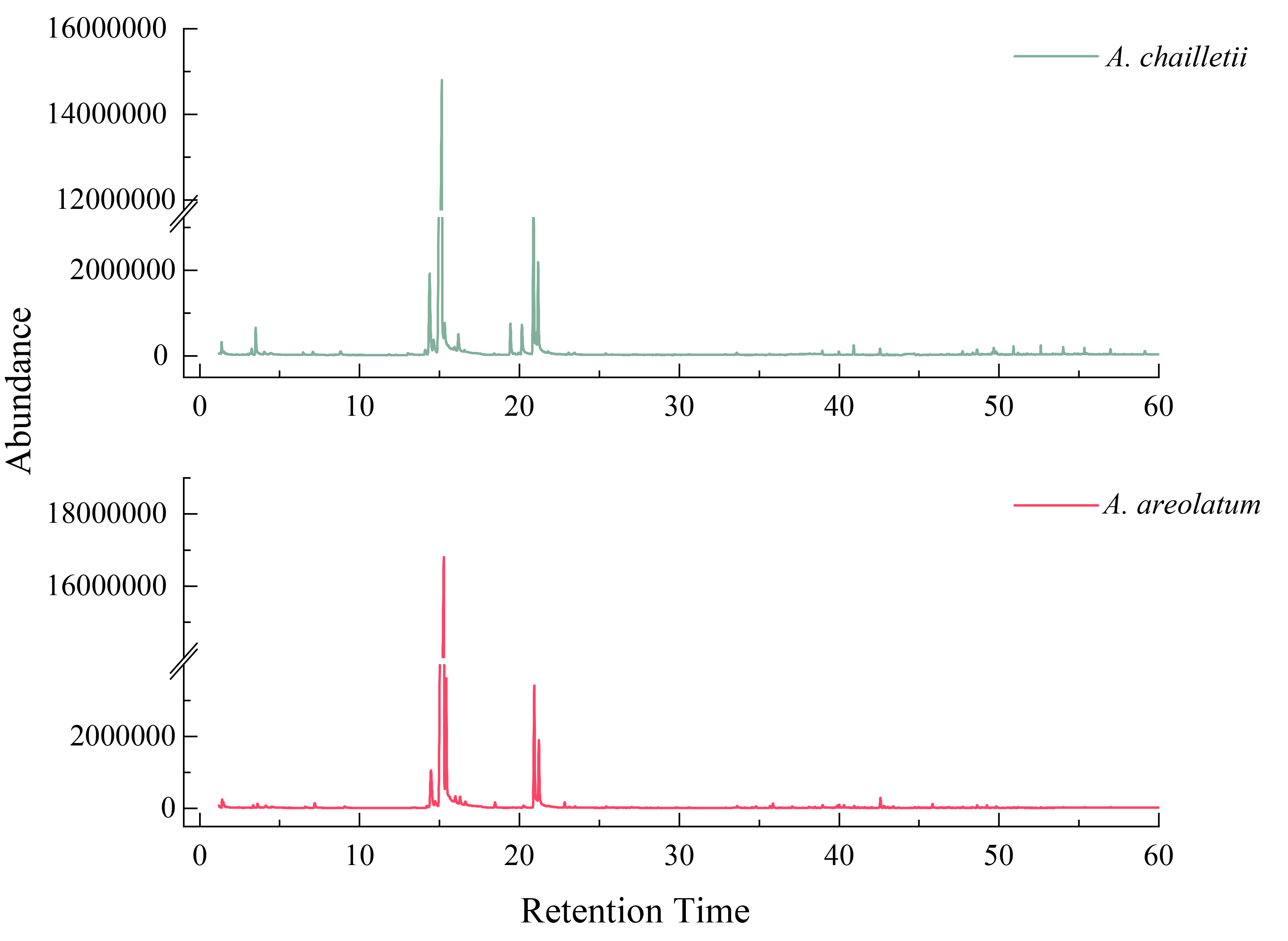


**Supplementary Figure 5 Extraction of volatile composition in *Amylostereum areolatum* and *A. chailletii* by Solid-phase microextraction**

## Supplementary Tables

**Supplementary Table 1.** **Movement parameters calculated by Track 3D**

| **parameter** | **Description/Formula** | **Example** |
| --- | --- | --- |
| Activation | Time to activation indicates time elapsed between the opening of the release cage and the emergence of the woodwasp. |  |
| Speed | The scalar, absolute value of the velocity vector in the 3D space.  *V_k_ is the velocity vector calculated for each sample s_k_.* | 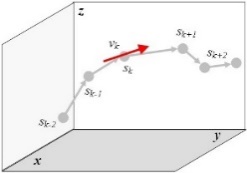 |
| Path 3d | The distance moved from the starting point of the present block^1^ up to the current sample^2^. It is measured as the sum of the segments joining adjacent samples and is expressed in mm.  *Dots are sample points in the 3D space. The sample s_0_ is the start of the block. Path 3d equals d_1_ for sample s_1_, d_1_+d_2_ for sample s_2_, d_1_+d_2_+d_3_ for sample s_3_, etc.* | *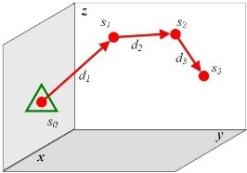* |
| Path x-y | The distance moved (in mm) up to the current sample projected on the x-y plane.  *Dark dots are the projection of the samples on the x-y plane. Path xy equals: d_1_ for sample s_1_, d_1_+d_2_ for sample s_2_.* *d_1_+d_2_+d_3_ for sample s_3_, etc.* |  |
| Path x-z | The distance moved (in mm) up to the current sample projected on the x-z plane.  *Dark dots are the projection of the samples on the x-z plane. Path xz equals:* *d1 for sample s_1_,* *d_1_+d_2_ for sample s_2_,* *d_1_+d_2_+d_3_ for sample s_3_, etc.* | ** |
| Tortuosity 3D | Equals the value of Path 3d divided by the length of the straight line connecting the starting sample of the block and the current sample.  Tortuosity 3d is a dimensionless number. It can only be equal to or larger than 1. When the samples in a block form a perfectly straight line, tortuosity is 1.  *l_3_ is the straight line connecting the starting sample of the block and sample s_3_.*  *Tortuosity 3d for sample s_3_= (d_1_+d_2_+d_3_)/ l_3_* | 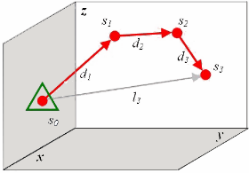 |
| Angular velocity 3D | Stands for Angular velocity 3d. It is the angular change 3d per time unit. It is expressed in degrees/s. |  |
| Angular change 3D | Stands for Angular change 3d. It is calculated as the angle in the 3D space formed by the segment joining s_k_ and s_k+1_ and the prolongation of the segment joining s_k_ and s_k-1_. *Angular change 3d* is expressed in degrees and can only be positive.  *Angular change 3d is the angle in the 3D space formed by the velocity vector and the prolongation of the previous velocity vector.* | 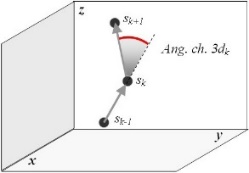 |
| Track xv | The angle in the 3D space formed by the velocity vector v_k_ (see Speed above) and the x axis.  *Example of Track xv. Note that the angle formed by the velocity vector and the x direction can lay on any plane passing by x, depending on the orientation of the vector.* |  |
| Course xv | It is calculated as Track xv, but considering the effect of air speed and direction. It is essentially the heading direction from the point of view of an observer who would move with the air. First consider the vector 'Heading relative to air', which is the 'intended' velocity and direction of movement in absence of wind. The actual heading is the resultant of this vector and the wind velocity vector.  *Course xv is the angle in the 3D space formed by the vector 'Heading relative to air' and the x direction.* |  |

1 Uninterrupted series of valid X, Y, Z data points

2 Set of X, Y, Z coordinates associated with a particular time value. Corresponds to a pair of X, Y coordinates in 2- D video tracking performed by EthoVision
